# Case Report: Neonatal heart failure: a rare presentation of congenital left ventricular aneurysm

**DOI:** 10.3389/fcvm.2026.1689014

**Published:** 2026-03-10

**Authors:** Aljuhara Alsuayyid, Mohammed Bin-Moallim, Omar Altamimi, Rimas Aloufi

**Affiliations:** Pediatric Cardiology, King Fahad Medical City, Riyadh, Saudi Arabia

**Keywords:** congenital heart disease, left ventricular aneurysm, neonatal heart failure, pediatric cardiology, pyruvate dehydrogenase deficiency

## Abstract

Congenital left ventricular aneurysm is a rare condition. Most patients remain asymptomatic for long periods. However, it carries a significant risk of serious complications, and management approaches are not well documented in the literature. We share our experience with an infant who had a positive genetic test indicating possible autosomal recessive pyruvate dehydrogenase complex deficiency with a non-specific cardiac association. The patient showed reduced cardiac output, received intensive treatment with antifailure medications, and was placed under close clinical observation, with nutritional improvement in preparation for surgical aneurysmectomy. Unfortunately, he was found dead at 6 months of age, possibly due to sudden cardiac death.

## Introduction

Congenital left ventricular aneurysm is a rare condition, with an estimated incidence of 0.5 per 10,000 live births (1), and it usually occurs in isolation. It is characterized by idiopathic endomyocardial dysplasia and should be distinguished from a congenital left ventricular diverticulum.

Generally, an aneurysm has a wide communication with the ventricle, whereas a diverticulum has a small or narrow communication with the ventricle (2). Based on the shape of the left ventricle, wall thickness, and the regional contractility of the bulging wall, Malakan Rad and coauthors proposed a classification system for congenital bulges (3):

− Type I: the left ventricle shows an abnormal shape, with preserved wall thickness and regional contractility at the site of the defect.
− Type IIa: the left ventricle shows an abnormal shape, with reduced wall thickness at the site of the defect.
− Type IIb: the left ventricle shows an abnormal shape, with reduced regional contractility of the defect wall.
− Type IIc: the left ventricle shows an abnormal shape, with reduced both wall thickness and regional contractility of the defect wall.

During embryogenesis, aneurysm formation is thought to be linked to localized myocardial ischemia. In 89% of cases, the wall of the anomaly is made of connective tissue, while in 11% of cases, all layers of the myocardium and pericardium remain intact. This structural abnormality can lead to severe hypokinesia or akinesia of the affected wall, along with asynchronous contraction. In contrast, the contractile ability of a diverticulum remains intact, and its size may stay the same or even decrease over time. Ventricular diverticula often occur alongside other congenital malformations, including defects of the sternum, pericardium, and diaphragm, as well as interventricular and atrial septal defects (4).

One of the most serious examples of this association is Pentalogy of Cantrell, which includes a group of thoracoabdominal wall defects characterized by cardiac ectopia and omphalocele. However, a left ventricular aneurysm typically appears as an isolated malformation (4). In Pentalogy of Cantrell, diverticula may also develop later in the course of the disease (5). A congenital left ventricular aneurysm or diverticulum can lead to a variety of clinical symptoms. However, 42% of patients with aneurysms and 63% of those with diverticula remain asymptomatic (4). Among symptomatic patients, congestive heart failure is common, reported in 71% of patients in the study by Papagiannis et al., as well as ventricular tachycardia or premature ventricular contractions (2).

A case involving an arrhythmogenic left ventricular apical congenital ventricular diverticulum in an adult has been reported. The condition was initially missed on echocardiography but was later detected by cardiac magnetic resonance imaging, showing its potential for unusual presentation and unexpected diagnosis (6).

In adults, an isolated congenital left ventricular diverticulum has also been associated with mitral valve infective endocarditis and an uncommon single coronary artery seen during surgery (7). Clinical symptoms may also involve thromboembolic events (8). Tilahun et al. described a deadly case of left ventricular aneurysm rupture that caused pericardial effusion in a child (9). In more than 50% of cases, these defects are identified incidentally during echocardiography, computed tomography, or magnetic resonance imaging (4).

## Case report

**Figure 1:**
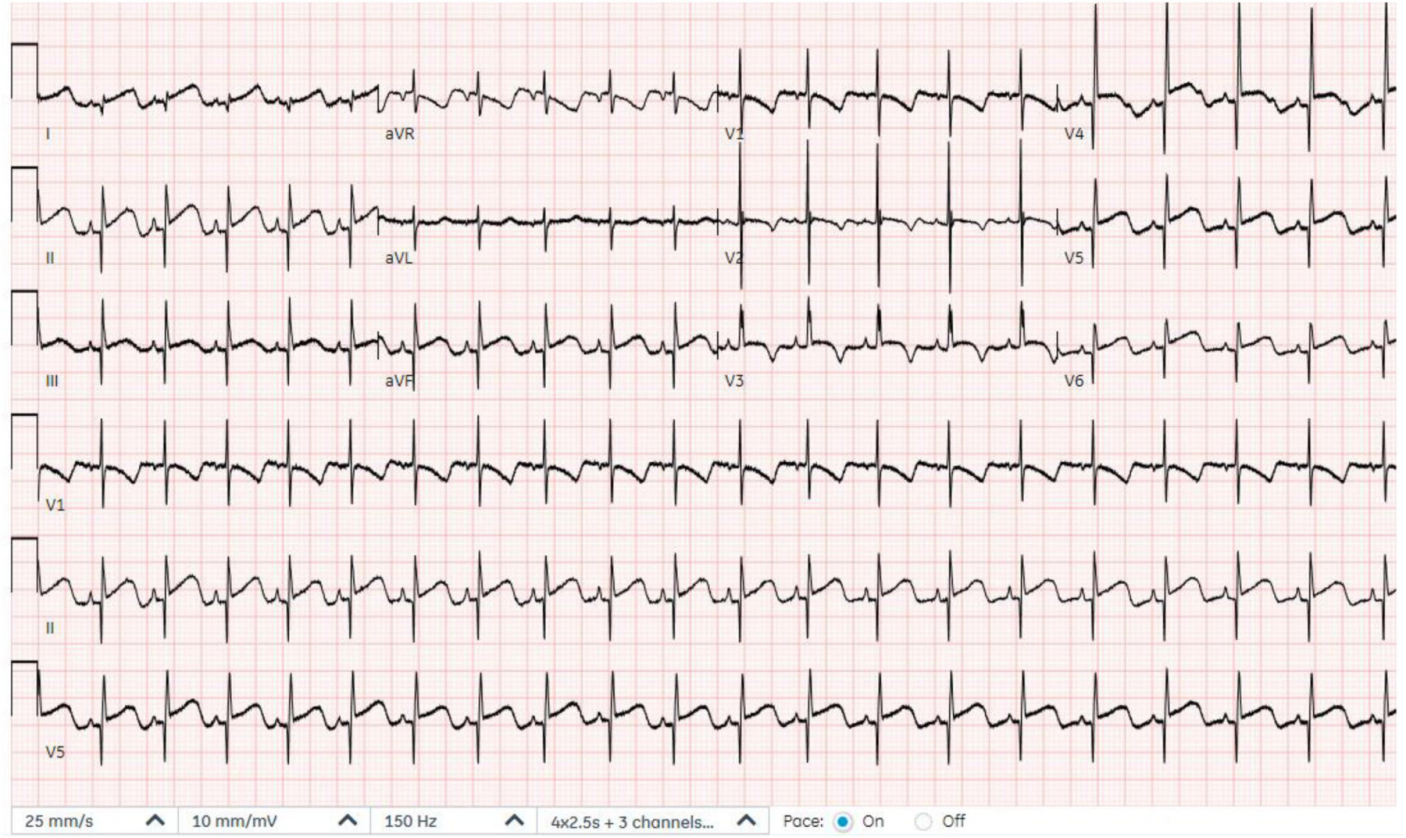
Electrocardiogram showing ST-segment elevation in lateral leads and deep Q waves.

**Figure 2:**
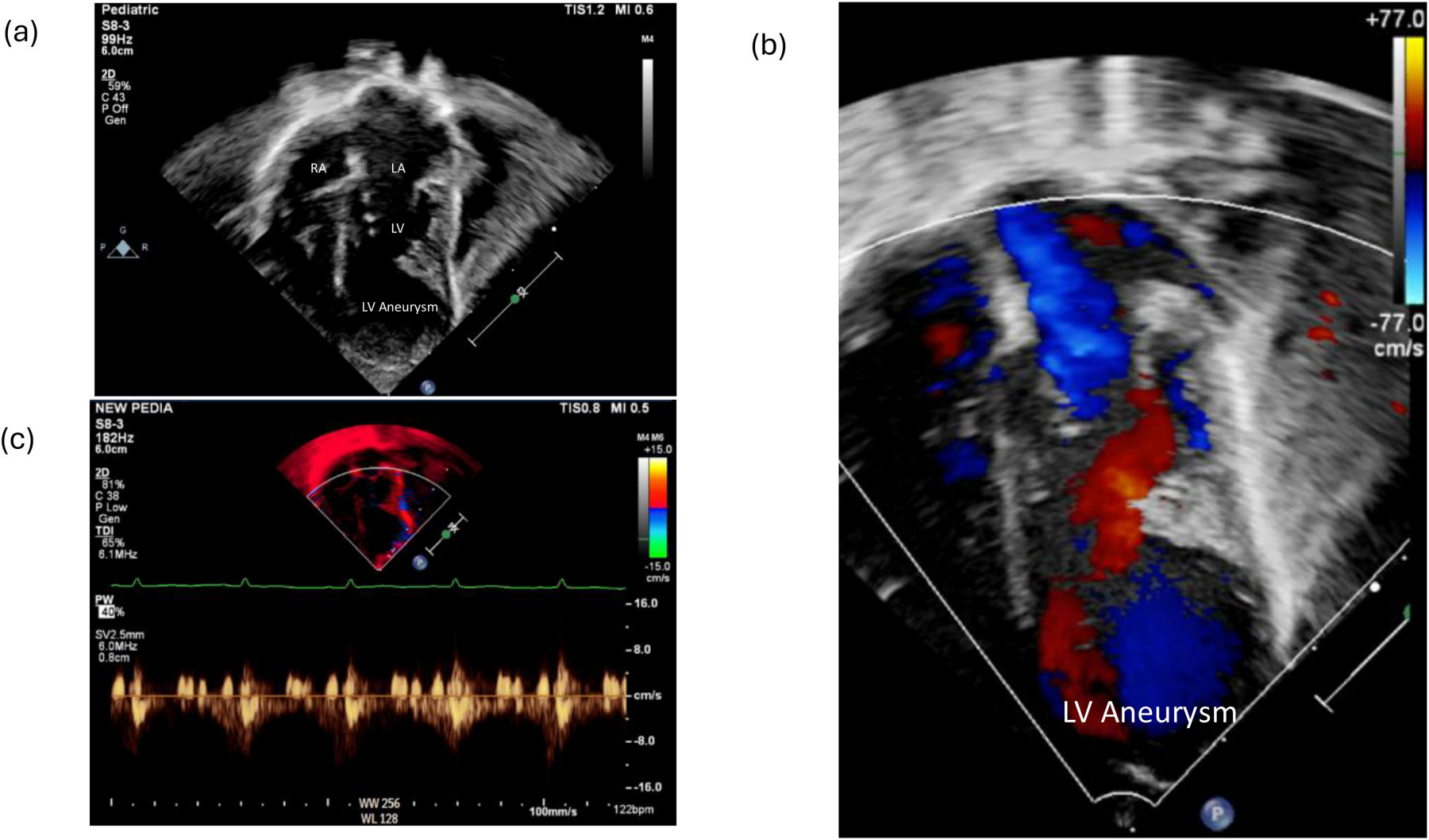
**(a)** Echocardiography showing a four-chamber view showing a left ventricular apical aneurysm. **(b)** Echocardiography showing a color Doppler two-chamber view showing a left ventricular apical aneurysm with swirling flow within the aneurysm. **(c)** Tissue Doppler echocardiography showing a left ventricular apical aneurysm with reduced lateral wall velocity. RA, right atrium; LA, left atrium; LV, left ventricle.

**Figure 3:**
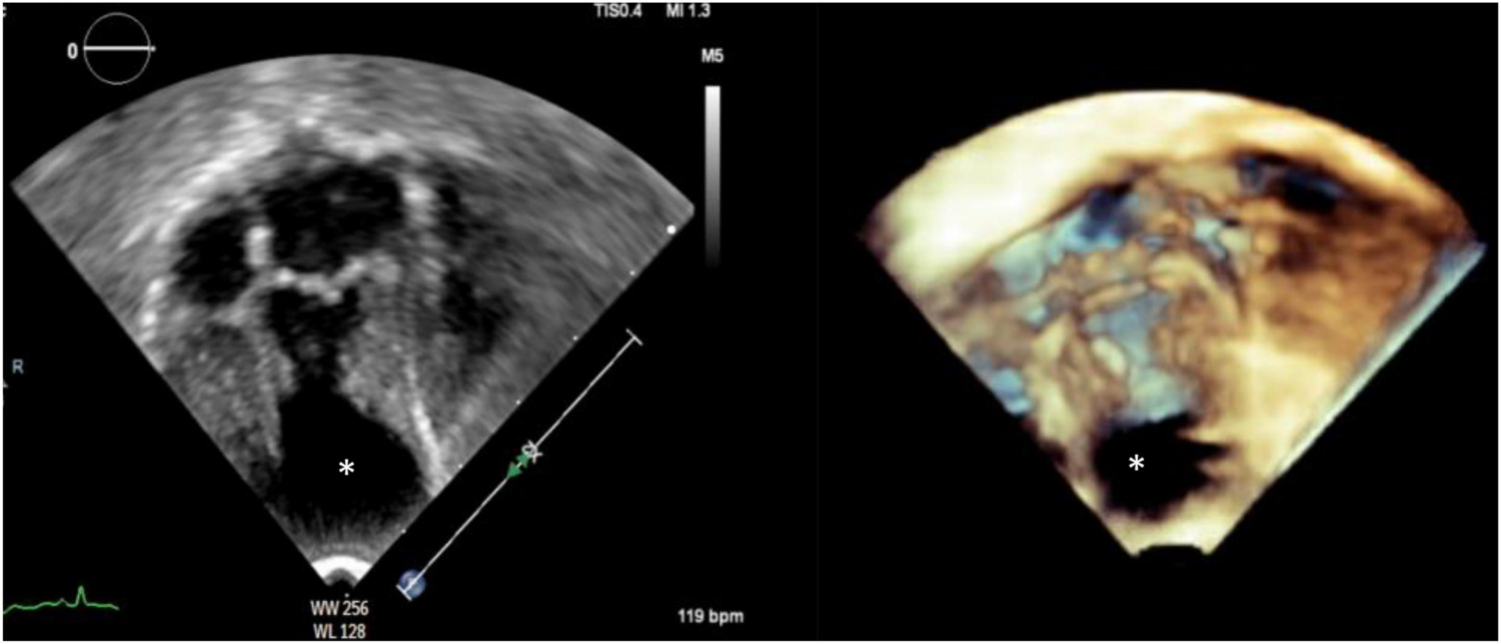
3D echocardiography showing an apical four-chamber view during systole. Dilated apical left ventricular aneurysm (asterisk) while the left ventricle is contracting (asynchrony).

**Figure 4:**
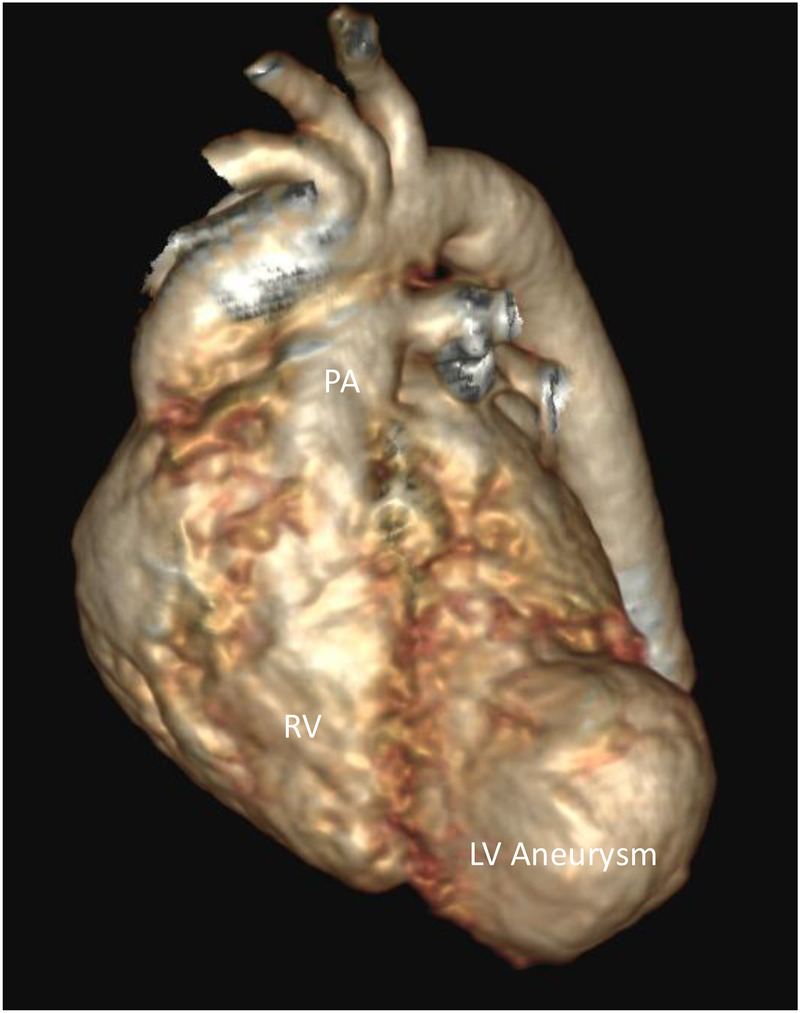
Constructed 3D cardiac CT showing a left ventricular apical aneurysm. RV, right ventricle; PA, pulmonary artery; LV, left ventricle.

A male infant was delivered at 36 weeks of gestation by cesarean section due to a previous maternal cesarean history. He was born to first-degree consanguineous parents and had a birth weight of 2 kg. A prenatal fetal echocardiogram at 29 weeks of gestation showed a cystic mass attached to the left ventricle. Further evaluation, including amniocentesis, fluorescence *in situ* hybridization (FISH), and chromosome sequencing, was negative for major known congenital anomalies. Apgar scores were 9 at both 1 and 5 min. At 2 h of age, the infant was noted to be tachypneic with increased work of breathing and was transferred to the neonatal intensive care unit, where he was placed on continuous cardiac monitoring and stabilized. On physical examination, oxygen saturation was normal on room air. No dysmorphic features were observed, and he had a grade 2/6 systolic murmur, best heard at the left lower sternal border. A chest X-ray obtained during the initial evaluation demonstrated mild cardiomegaly. Basic septic and electrolyte studies were unremarkable. An electrocardiogram showed ST-segment elevation in the lateral leads, along with deep Q waves, indicating ischemic changes (Figure 1). The cardiology team was involved to evaluate the heart murmur and rule out cardiac causes. Echocardiography revealed a small atrial septal defect and a large left ventricular apical aneurysmal dilatation with hypokinetic regional wall motion (Figures 2a,b). No abnormal coronaries were identified. Tissue Doppler echocardiography showed reduced velocities in both the lateral and medial walls of the aneurysm (Figure 2c). Three-dimensional echocardiography revealed a dilated apical left ventricular aneurysm during systole, indicating asynchrony with the adjacent left ventricular myocardium (Figure 3). Cardiac CT showed a large aneurysmal dilatation arising from the left ventricular apex, with a wide neck (Figure 4).

At 3 weeks of age, while admitted to our unit for further evaluation, the patient was noted to have poor feeding, reduced urine output, rapid heart rate, and low blood pressure. These findings were consistent with low cardiac output and suggestive of cardiac failure. His NT-proBNP level was markedly elevated at 4,528 pg/mL (normal <300 pg/mL), along with evidence of metabolic acidosis and a high lactate level of 7.5 mmol/L (normal 0.56–1.39 mmol/L).

He was treated aggressively for heart failure with medications, including furosemide and captopril. Aspirin was briefly initiated to prevent potential ventricular thrombosis; however, this was excluded by echocardiography, and aspirin was subsequently discontinued. His C-reactive protein (CRP) was elevated at 33.3 mg/L (normal <5.0 mg/L), and empirical antibiotic therapy with ampicillin and cefotaxime was started while a full septic workup was performed. Blood cultures later grew *Candida parapsilosis*, prompting discontinuation of antibiotics and initiation of antifungal therapy with AmBisome (amphotericin B) for a 6-week course.

Given the persistently elevated lactate levels, an underlying metabolic disorder was considered; however, no definitive cause was identified other than features consistent with low cardiac output. Repeated blood cultures were negative, and his clinical condition improved significantly. Over time, he demonstrated gradual clinical improvement with resolution of acidosis, although lactate levels remained persistently elevated (3.9–10 mmol/L).

During his hospital stay, he underwent a comprehensive evaluation by the neurology and metabolic teams. The only significant finding was a positive genetic test, suggesting possible autosomal recessive pyruvate dehydrogenase complex deficiency (PDCD), a condition known to be associated with elevated lactate levels.

**Figure 5:**
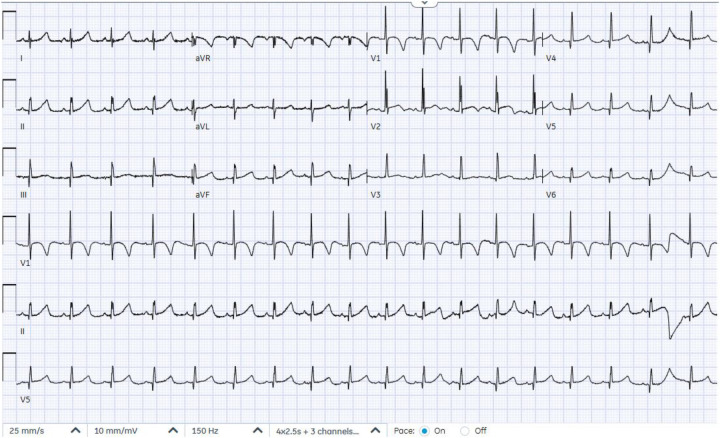
Electrocardiogram showing normal sinus rhythm and no ischemic signs.

The patient remained clinically stable, with good feeding, appropriate weight gain, and stable vital signs. Repeated electrocardiography demonstrated sinus rhythm without ST-segment elevation (Figure 5). Follow-up echocardiography showed no interval change in the large left ventricular apical aneurysmal dilatation, with persistent regional wall motion hypokinesia. The management plan included continued outpatient follow-up to allow further growth before a planned surgical aneurysmectomy. Sadly, at 6 months of age, he was found deceased, likely due to sudden cardiac death.

## Discussion

Congenital left ventricular aneurysm and diverticulum are rare cardiac malformations characterized by focal ventricular outpouching; the actual prevalence is thought to be much higher since many of them are asymptomatic. Fewer than 1,000 cases have been reported so far; thus, a differentiation between these two conditions, given their overlapping morphological and functional characteristics, remains difficult, despite advances in cardiac imaging modalities. Diverticula usually arise from delayed myocardial development. They typically include all layers of the myocardium, demonstrate preserved synchronous contractility, and are often part of other congenital anomalies. In contrast, an aneurysm likely results from ischemia of the embryonic myocardium, is usually composed of fibrous tissue, exhibits hypokinesia or akinesia, has a broad base, and may progressively dilate. Unlike diverticula, aneurysms often occur as isolated defects. Overlapping morphological and functional features often complicate diagnosis (4). Malakan Rad et al. proposed a geometry-based classification system for congenital left ventricular bulges based on ventricular shape, wall thickness, and regional contractility to improve the classification and guide management: Type I, shape abnormality with preserved wall thickness and contractility; Type IIa, shape abnormality with reduced wall thickness; Type IIb, shape abnormality with reduced regional contractility; and Type IIc, shape abnormality with both reduced wall thickness and contractility (3).

In our patient, the left ventricular outpouching was clearly visible in the apical region; it had a wide neck with free blood flow into the cavity, along with hypokinesia and asynchronous regional motion of the lower apical segment. These findings supported the diagnosis of a ventricular aneurysm. Echocardiography is a widely available, non-invasive, and highly informative diagnostic modality for evaluating congenital left ventricular aneurysms and diverticula, which allows accurate measurement of outpouching size, evaluation of wall contractility, assessment of ventricular function, and identification of associated cardiac anomalies. However, a congenital ventricular aneurysm is a rare condition and may be missed due to difficulty in adequately visualizing and properly aligning the apical region during echocardiographic examination (10).

In our patient, the coronary artery connections and courses were normal. However, in a study of 117 patients, coronary anomalies were present in 58.1% of cases, most frequently in men and in those with left ventricular aneurysm or diverticulum. During a 4.2-year follow-up, the incidence of adverse cardiac events did not differ between patients with and without coronary anomalies, and no life-threatening coronary abnormalities were found. These results indicate that abnormal coronary anatomy is a common feature in isolated LVA or LVD, occurring in more than half of affected patients (11). These defects may present with a broad spectrum of clinical manifestations; however, a significant number of patients may remain asymptomatic: 42% of those with aneurysms and 63% of those with diverticula. Serious complications during childhood are rare, and most cases are diagnosed incidentally during cardiac imaging. In our patient, genetic investigation revealed a homozygous variant of uncertain significance in the PDHB gene, suggestive of a possible PDCD, a mitochondrial disorder that impairs carbohydrate metabolism, reduces ATP production, and primarily affects the brain. PDCD usually presents in early childhood with developmental delay, neurological manifestations such as hypotonia, epilepsy, ataxia, and microcephaly, characteristic brain abnormalities, and metabolic disturbances like lactic acidosis and elevated pyruvate levels. The age at presentation varies, with a median age at diagnosis of approximately 20 months (12). Until now, no case reports or research findings have clearly established a direct link between left ventricular aneurysm in childhood and recessive pyruvate dehydrogenase E1-beta deficiency; however, such a potential link could be considered in our patient. He developed low cardiac output that responded to antifailure medication, including furosemide and captopril, resulting in clinical improvement. Notably, heart failure has been repeatedly reported as resistant to medical therapy and represents a major cause of death in these patients (2).

The management remains debatable between conservative and surgical intervention; however, interventional device closure of the aneurysm has also been reported (13). In our patient, we initiated treatment with antifailure medications and planned for surgical intervention; however, his body weight precluded the plan. As there is no clearly established weight threshold, we elected to observe him clinically and accepted a weight of 3 kg for intervention, unless clinical concern arose during observation.

The prognosis and long-term outcomes vary widely depending on the type of bulge. It was found that within 8.5 months of birth, 40% of children with left ventricular aneurysm developed complications. In contrast, no serious complications were observed during a 10-year follow-up of children with diverticula. In general, the prognosis for these defects is favorable, and many children remain asymptomatic for a long time. Nevertheless, there is a risk of the development of severe complications, such as aneurysm rupture, thrombosis, embolism, pericardial effusion, and death. Mortality has been reported in 4% of patients younger than 18 years of age (4).

## Data Availability

The original contributions presented in the study are included in the article/Supplementary Material, further inquiries can be directed to the corresponding author.
